# Case Report: A Virilizing Adrenal Oncocytoma

**DOI:** 10.3389/fsurg.2021.646459

**Published:** 2021-03-22

**Authors:** Efstathios Kotidis, Stefanos Bitsianis, Konstantinos Galanos-Demiris, Panagiotis Christidis, Ioannis Mantzoros, Orestis Ioannidis, Vasilis Foutsitzis, Manousos George Pramateftakis, Stamatios Aggelopoulos

**Affiliations:** Fourth Academic Department of Surgery, Faculty of Health Sciences, School of Medicine, Aristotle University of Thessaloniki, Thessaloniki, Greece

**Keywords:** adrenal mass, testosterone, masculinization, virilization, borderline, oncocytoma

## Abstract

A 64-year-old female was admitted to our clinic with a 9-cm-sized adrenal mass. The patient's main symptom was hirsutism, which included thinning scalp hair and excessive hair growth over her torso and arms. Upon investigation, elevated values of testosterone, androsterone D4, and DHEA-S were found. Contrast-enhanced abdominal CT and MRI scans revealed a heterogenous large mass (diameter 9 × 8.5 cm) with focal calcifications, necrotic areas, and a clear distinction from the adjacent structures. The patient underwent a right adrenalectomy. The histological examination of the tumor revealed a borderline adrenocortical oncocytoma. The patient had an uncomplicated postoperative course and was discharged on postoperative day 8. Similar cases reported in the literature are also being reviewed in this case report.

## Introduction

Oncocytomas or oncocytic neoplasms are tumors composed exclusively or predominantly of cells called oncocytes or oncocytic cells (1). Oncocytes were first described by Hamperl in 1931 as large, highly eosinophilic, granular cells typically associated with a Hurthle cell tumor of the thyroid gland (2). Ultrastructurally, granular cytoplasm is caused by the massive accumulation of mitochondria (3). Oncocytomas arise most frequently in the kidney, salivary glands, thyroid gland, parathyroid, and pituitary gland (1).

Adrenal oncocytomas are an extremely rare entity, and since their first description by Kakimoto et al. in 1986, a total of ~185 cases have been reported worldwide (4, 5). They account for 1.8% of adrenal tumors and are usually detected incidentally, presented as nonfunctional and benign tumors (6). However, recent data suggest that about 20% of adrenal oncocytomas are malignant according to the criteria proposed in 2004 by Bisceglia et al. and about 30% are functional, secreting corticosteroids, androgens, aldosterone, estrogens, catecholamines, or a combination of these and cause symptoms attributed to these hormones (5, 7).

The aim of our paper is to report a case of a virilizing androgen-secreting adrenal oncocytoma and provide a summary of the very limited literature regarding the clinical and pathological features of similar cases.

## Case Description

A 64-year-old Caucasian female was referred to our clinic by her private doctor for a mass in the region of her right adrenal gland, ~9 cm in size. The mass was found, accidentally, during an ultrasonography for known gallstones. The patient did not complain about abdominal pain or discomfort. On clinical examination, a mass was palpated in the right renal region. Hirsutism, with thinning scalp hair and excessive hair growth over her torso and arms, was noted. However, our patient did not report any other symptoms such as weight loss, headaches, diaphoresis, palpitations, or any change in her skin, urination, and bowel habits.

Her past medical history included arterial hypertension, treated with nebivolol, amlodipine, and valsartan, and two surgical operations (hysterectomy due to uterine fibroids, 5 years ago, and an excision of a right breast fibroadenoma, 30 years ago).

The patient underwent full laboratory testing, useful for the differential diagnosis of the mass, which showed elevated values of testosterone (1.6 ng/mL, normal 0.1–0.9 ng/mL), androsterone D4 (5.1 ng/mL, normal 0.3–3.3 ng/mL), and DHEA-S (3.4 μmol/L, normal 0.9–2.1 μmol/L). The values of cortisol, aldosterone, dopamine, epinephrine, norepinephrine, and creatinine were within normal ranges.

**Figure 1:**
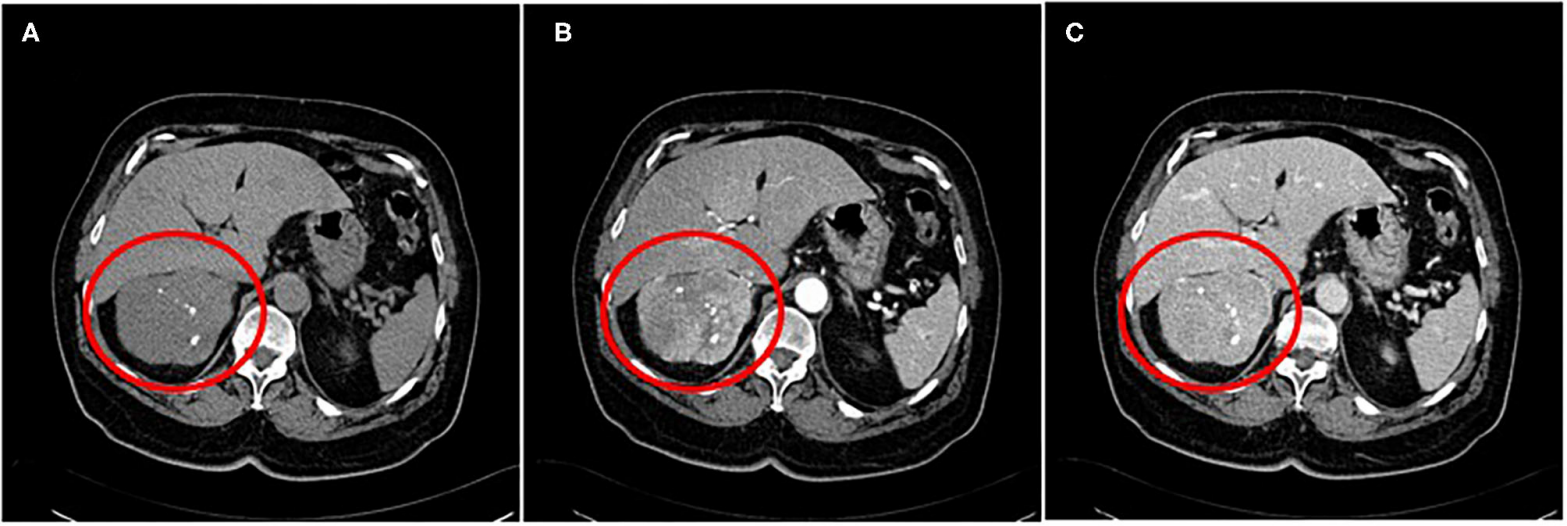
A contrast-enhanced abdominal CT scan showing a quite large heterogenous mass with focal calcifications **(A)** ~9 cm in size. The lesion had not only arterial **(B)** but also venous enhancement, while some areas remained nonenhanced, possibly necrotic **(C)**.

A contrast-enhanced abdominal CT scan showed a quite large heterogenous mass (diameter 9 × 8.5 cm) with focal calcifications (Figure 1). The lesion had not only arterial but also venous enhancement, while some areas remained nonenhanced, possibly necrotic. The blood flow through the inferior vena cava was normal, and no enlarged lymph nodes were spotted. However, two lesions on the left liver lobe (9 and 19 mm) and one on the right liver lobe (12 mm) with high blood supply were identified. Chest CT scan was clear for any suspicious lesions. Cancer markers CA19-9, CEA, and CA125 were measured and were all within normal values.

**Figure 2:**
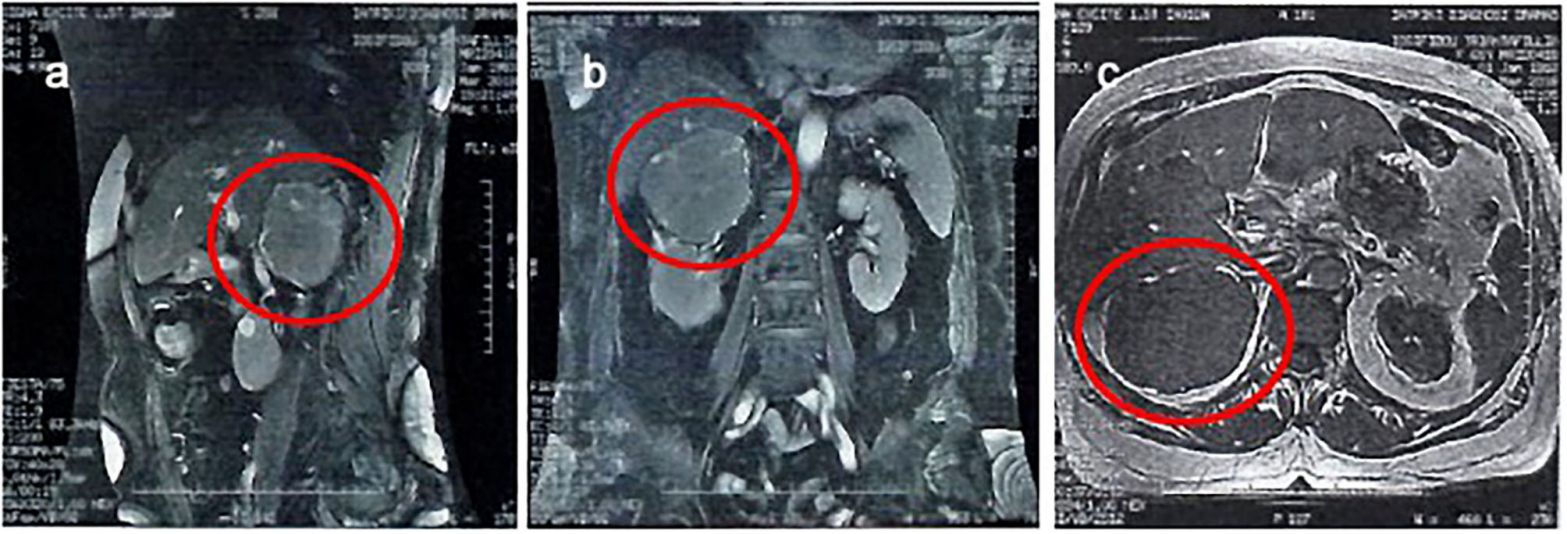
An abdominal MRI scan was performed, and it showed the right adrenal gland lesion. It also showed a clear distinction between the lesion and adjacent structures, without any infiltration to the inferior vena cava, or presence of pathological lymph nodes, nor were there any pathological lesions or enhancement in the liver. A sagittal T2 view **(a)**, a coronal T2 view **(b)**, and a transverse T1 view **(c)**.

An abdominal MRI scan was performed, and it showed the right adrenal gland lesion, with a mean signal intensity in the T1W images, and a mean signal (with a few high-intensity elements) in the T2W images (Figure 2). It also showed a clear distinction between the lesion and adjacent structures, without any infiltration to the inferior vena cava, or presence of pathological lymph nodes, nor were there any pathological lesions or enhancement in the liver.

Taking into consideration the tumor size, the secretion of androgens, and the possibility of malignancy, an open surgical approach was decided. The patient underwent a trans-abdominal right lateral adrenalectomy. No drugs were administered preoperatively, as no clinical or biochemical findings of cortisol or catecholamine hypersecretion were found.

**Figure 3:**
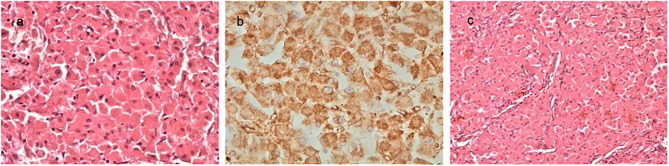
The histological examination of the tumor revealed a borderline adrenocortical oncocytoma. Macroscopically, the tumor was a descriptive almost circular lesion, surrounded by a fibrous capsule. Its size was 9.4 × 8.5 × 7.3 cm, and it weighted 113 g. Microscopically, there were large round eosinophilic cells (oncocytes) with dense granular cytoplasm; nuclei were round and regular with even chromatin; small but conspicuous nucleoli were present (**a**, H&EX40). Immunohistochemical tests revealed that the tumor cells were positive for vimentin (**b**, ×40), CD56, Melan A, S-100, and synaptophysin. Oncocytoma-compact nested architecture, uniform round nuclei, and abundant pink cytoplasm (**c**, H&E ×40).

The histological examination of the tumor revealed a borderline adrenocortical oncocytoma. Macroscopically, the tumor was a descriptive, almost circular lesion, surrounded by a fibrous capsule. Its size was 9.4 × 8.5 × 7.3 cm, and it weighted 213 g. In its cross sections, it had a white-yellow complexion, had a coarse nodular shape, and it was elastic. In places it had a soft and focal chalky texture. Microscopically, the tumor emerged from large cells arranged in nests or beams, with dense eosinophilic cytoplasm and small round deep-colored nuclei, without increased nuclear atypia or mitotic activity. Finally, immunohistochemical tests revealed that the tumor cells were positive for vimentin, CD56, Melan A, S-100, and synaptophysin (Figure 3).

The patient had an uncomplicated postoperative course and was discharged on the 8th postoperative day. As the tumor was not malignant, she received no further treatment. The timeline of the patient's course is presented in Table 1. However, she was reevaluated clinically and with an ultrasound or MRI scan every 6 months for the first 2 years and then annually with an MRI until the completion of 5 years. Hirsutism was resolved during her first follow-up meeting. Currently, she is in the 2-year interval after the resection of the mass without any sign of recurrence.

**Table 1:** Abbreviated presentation of the patient's course.

1	Admission	Hirsutism and palpable abdominal mass
2	Random artifact	Ultrasonography: tumor of ~9 cm in diameter in the right adrenal gland
3	Investigation	Laboratory tests: Testosterone: 1.6 ng/mL Androsterone D4: 5.1 mg/mL DHEA-S: 3.4 μmol/mL Imaging (CT and MRI scan)
4	Treatment	• Trans-abdominal right lateral adrenalectomy • Histological examination
5	Post-operative course	• Uncomplicated course • Discharged on postoperative day 8.

## Discussion

Adrenal oncocytomas are a rare form of adrenal tumors, and the functional ones are a small minority of these. More specifically, functional oncocytomas responsible for the masculinization of female patients due to the increased androgen secretion occur even less frequently. We identified 19 such cases from literature, of which we were able to retrieve 17 and collect clinical, biochemical, and histological data (Table 2) (8–22, 24–26).

**Table 2:** Clinical data of the patients.

	**Age, y**	**Presentation**	**Hormones**	**Tumor size, cm**	**Tumor weight, g**	**Site**	**Histological diagnosis**	**Immunohistochemical studies**
Logasundaram et al. (8)	58	Hirsutism, Cushing	Androgen, cortisol	8.8	340	R	Oncocytoma benign	Cytokeratin, synaptophysin, vimentin, Melan-A
Geramizadeh et al. (9)	43	Hirsutism, Cushing	Androgen, cortisol	9	195	L	Oncocytoma benign	Synaptophysin
Lim et al. (10)	14	Virilization	Androgen	17.5	1,100	R	Oncocytoma benign	Unknown
Mwandila et al. (11)	19	Hirsutism	Androgen	5	67	L	Oncocytoma malignant	Unknown
Wong et al. (12)	53	Virilization	Androgen	13	670	L	Oncocytoma malignant	Vimentin, synaptophysin, Melan-A, inhibin-A, calretinin, mES-13
	41	Virilization	Androgen	28.5	5,720	L	Oncocytoma malignant	Synaptophysin, Melan-A, inhibin-A, calretinin
Sharma et al. (13)	16	Virilization	Androgen	12	Unknown	R	Oncocytoma benign	Unknown
Surrey et al. (14)	55	Hirsutism	Androgen	7	55.5	Ectopic	Oncocytoma benign	Synaptophysin, Melan-A, inhibin-A, calretinin
Subbiah et al. (15)	3.5	Virilization	Androgen	2.5	20	R	Oncocytoma benign	Unknown
Sahin et al. (16)	23	Hirsutism	Androgen	2.2	Unknown	L	Oncocytoma benign	CD56, synaptophysin, vimentin, Melan-A
Tetsi Nomigni et al. (17)	34	Hirsutism, spaniomenorrhea	Androgen	2.6	Unknown	R	Oncocytoma benign	Unknown
Yordanova et al. (18)	9	Virilization	Androgen	Unknown	Unknown	R	Oncocytoma benign	Unknown
Carré et al. (19)	50	Virilization	Androgen	3	Unknown	L	Oncocytoma malignant	Unknown
Liu et al. (20)	12	Hirsutism, Cushing	Androgen, cortisol	2.5	8	Ectopic	Oncocytoma benign	CD56, synaptophysin, vimentin, Melan-A, inhibin-A, NSE, pancytokeratin
Ramareddy et al. (21)	11	Virilization, hirsutism	Androgen	6	Unknown	L	Oncocytoma borderline	Synaptophysin, vimentin, Melan-A, cytokeratin
Hong et al. (22)	36	Virilization	Androgen	3	Unknown	L	Oncocytoma benign	Vimentin, inhibin-A
Bisceglia et al. (23)	24	Virilization	–	20	1,400	R	Oncocytoma borderline	Keratin, vimentin, Melan-A, mES-13

*R, right; L, left; y, years; cm, centimeters; g, grams*.

These tumors appear in females, as was our patient, and can occur in a large age range (3.5–58). Their clinical signs are vocal changes, muscle hypertrophy, breast atrophy, and clitoral hypertrophy, or simply hirsutism with thinning scalp hair and excessive hair growth. In three out of the 19 cases, features of Cushing's disease were present (8, 9, 20). Elevated androgen secretion (testosterone and DHEA) was found in all cases explored in the reported literature. In our case, testosterone, androsterone D4, and DHEA-S were elevated, albeit all other hormones and cancer markers (CA19-9, CEA, and C125) were normal. Patients with Cushing's disease also presented high blood cortisol values, while similarly high cortisol values were also present in a female with signs of masculinization, but without Cushing's disease (10). There are no specific findings that can lead to a certain diagnosis through a CT or MRI scan. They usually have a noninvasive image with a CT value of 20–40 HU and a heterogeneous radiographic contrast agent uptake (27).

Tumor size ranged from 2.2 to 28.5 cm and tumor weight ranged from 8 to 5,720 g. In terms of their localization, they were all unilateral, found in the anatomical position of the right (41.2%, 7/17) or left (47.0%, 8/17) adrenal gland, with the exception of two cases (11.8%, 2/17), where these tumors were identified in an ectopic retroperitoneal position (14, 20). Macroscopically, most tumors were clearly marginated, were subcircular, had a fibrous capsule, and were white, yellow, or brown in the cross section.

Microscopically, they were composed of large eosinophilic and granular cytoplasmic cells with a central pycnotic nucleus, with a solid, trabecular, tubular, or papillary growth pattern. Finally, regarding the immunohistochemistry of the oncocytomas, they appear to be positive for vimentin, Melan-A, synaptophysin, and inhibin-α and negative for chromogranin. Regarding the percentage of malignancy among masculinizing oncocytomas reported in the current literature, it is similar to that of oncocytomas in general (~20%). However, there are specific criteria for the characterization of adrenal oncocytomas (benign, malignant, or borderline) as defined in 2004 by Bisceglia et al. (23). The major criteria are venous invasion, presence of atypical mitoses, and a high mitotic rate (more than five mitoses per 50 high-power fields) while the presence of necrosis, size more than 10 cm, weight more than 200 g, and capsular invasion or sinusoidal invasion are considered as minor criteria. The presence of at least one of the major criteria is indicative of malignancy, while the presence of one of the minor criteria is indicative of a borderline oncocytoma (23). Our case was classified as a borderline or as of uncertain malignant potential, since only one minor (weight 213>200gr) criterion of the Bisceglia criteria was fulfilled from the histological examination.

As the final diagnosis of these adrenal masses is based primarily on histological examination and immunohistochemistry, their management should be surgical removal (28). Size and imaging characteristics are the main factors that guide the clinician to this decision (29). Although <2% of adrenal tumors/incidentalomas with a size smaller than 4 cm represent primary adrenal carcinomas, the risk for adrenal carcinoma increases to 25% in adrenal masses >6 cm (30). In addition, several imaging features should increase the suspicion of malignancy: tumor size, irregular margins, central intratumoral necrosis or hemorrhage, heterogeneous enhancement, invasion into adjacent structures, and calcification (31).

Regarding the surgical approach that should be performed (open or laparoscopic), this also depends on their size and function (28). Traditionally, large tumors, bigger than 6 cm and with a high suspicion of malignancy, are removed with an open surgical procedure. This recommendation is still valid in the recent guidelines for adrenal tumors by the National Comprehensive Cancer Network (NCCN) and European Society of Medical Oncology (ESMO) (32). However, there are studies that demonstrate the safety of laparoscopic adrenalectomies, even for tumors with these characteristics (33). We opted for an open approach, with a trans-abdominal right lateral adrenalectomy, due to the tumor size and the overall malignancy possibility. Although the laparoscopic experience of our team on adrenalectomies could support the laparoscopic technique, we preferred this approach, so as to achieve a precise, en bloc, tumor resection.

The rarity of these tumors and the lack of wide clinical data do not allow the establishment of guidelines for the postoperative treatment and follow-up of oncocytomas. However, the data so far show that benign adrenal tumors have an excellent prognosis, with only one case reported in the literature as recurrent (23). The same seems to be true for borderline oncocytomas, where recurrence was observed in just one publication (12). Wong et al. also reported 8% relapse/metastasis and 3% tumor-related death (12). Finally, in terms of malignant tumor survival, it exceeds that of adrenal cortical carcinomas reaching up to 58 months (12). In this context, we included the patient in a follow-up program with clinical and hormonal examination every 6 months as well as imaging with u/s, MRI, etc.

To conclude, masculinizing adrenal oncocytomas are extremely rare tumors, characterized by androgen hypersecretion and clinical characteristics resulting from androgen hypersecretion. Surgical resection remains the recommended treatment and the only one that will lead, with the help of an experienced pathologist, to a safe diagnosis of the disease, and its categorization as benign, borderline, or malignant.

## CARE Checklist (2016) Statement

The authors have read the CARE Checklist (2016), and the manuscript was prepared and revised according to the CARE Checklist (2016).

### Patient Perspective

The patient was informed that her name and initials will not be published and that efforts will be made to conceal her identity, while images or other clinical information relating to her case may be presented in a medical publication and as a result the material may be seen by the general public.

## Data Availability Statement

The original contributions presented in the study are included in the article/supplementary material, further inquiries can be directed to the corresponding author/s.

## Ethics Statement

Ethical review and approval was not required for the study on human participants in accordance with the local legislation and institutional requirements. The patients/participants provided their written informed consent to participate in this study.

## Author Contributions

EK, SB, and PC conceived the paper's objective and performed data collection and extraction. EK, SB, OI, and KG-D performed the operations and were responsible for the postoperative course of the patient. All aforementioned authors along with MP and VF wrote the initial draft. IM and SA offered significant help in revising the present manuscript in order to reach its definitive form. All authors made substantial contribution and reviewed the document carefully prior to submission.

## Conflict of Interest

The authors declare that the research was conducted in the absence of any commercial or financial relationships that could be construed as a potential conflict of interest.
